# ZnO/Boron Nitride Quantum Dots Nanocomposites for the Enhanced Photocatalytic Degradation of Methylene Blue and Methyl Orange

**DOI:** 10.3390/molecules27206833

**Published:** 2022-10-12

**Authors:** Di Liu, Jinu Song, Jin Suk Chung, Seung Hyun Hur, Won Mook Choi

**Affiliations:** School of Chemical Engineering, University of Ulsan, 93 Daehak-ro Nam-gu, Ulsan 44610, Korea

**Keywords:** ZnO, boron nitride quantum dots, methylene blue, methyl orange, photocatalyst

## Abstract

In this study, a heterostructure photocatalyst of ZnO nanoparticles decorated with boron nitride quantum dots (ZnO/BNQDs) was successfully synthesized by a simple solution procedure. The synthesized ZnO/BNQDs show that the BNQDs effectively suppress the recombination of photoinduced electrons and holes and the transfer of holes from ZnO nanoparticles by the formation of a heterojunction. The ZnO/BNQD nanocomposites thus demonstrate superior photocatalytic performances and excellent stability for the degradation of methylene blue (MB) and methyl orange (MO) under UV light irradiation. Based on the obtained results, the possible photocatalytic mechanism is proposed and discussed. Thus, the ZnO/BNQD nanocomposites demonstrate potential as an efficient low-cost photocatalyst for application in the photodegradation of organic dyes in wastewater for environmental remediation.

## 1. Introduction

The remediation of water pollution has attracted considerable attention due to the increasingly serious environmental problem presented by rapid industrialization with numerous pollutants, such as dyes, organic solvents, and oil [1,2]. Among various technologies for the treatment of water pollution, the photocatalytic approach using semiconductor materials with light has been an area of considerable interest as a versatile and environmentally friendly technique, because of its high efficiency, low cost, easy preparation of catalysts, and absence of pollution byproducts [3,4,5,6,7,8]. For semiconductor photocatalysts, ZnO has been widely explored for the remediation of water, due to its high photosensitivity, low cost, and excellent chemical and physical stability [9,10,11,12,13]. However, the photocatalytic efficiency of ZnO is greatly limited by the narrow light-absorption range and fast recombination of photoinduced electron-hole pairs [14,15,16]. Therefore, various strategies have been developed to overcome these disadvantages. One of the effective methods is the heterojunction formation with two-dimensional nanomaterials, which suppresses the recombination of photoinduced electron–hole pairs [17,18,19,20,21,22]. Atchudan et al. [23] synthesized the hybrid of ZnO on an N-doped graphitic carbon sheet, which demonstrated that the N-doped graphitic carbon enhanced the light absorption and reduced the charge recombination, resulting in enhanced photocatalytic efficiency. Similarly, Sindhuja et al. [24] fabricated the composites of ZnO/g-C_3_N_4_/Ag via a spray technique and reported the enhanced photodegradation performance for methylene blue (MB) and malachite green (MG) dyes.

Graphene-like boron nitride (BN), known as “white graphene”, possesses unique properties that include good optical characteristics, low toxicity, high thermal conductivity, and fast charge transfer, which in recent years has attracted a great deal of attention [17,18,19,20,21,22,23,24,25,26,27,28]. In addition, boron nitride quantum dots (BNQDs) are a new class of boron nitride derivatives with fascinating fluorescence properties and excellent water dispersibility due to the large edge effects and defects on the boron nitride lattice. Therefore, BNQDs are explored to attract photoinduced holes and boost the spatial separation of photoinduced electron–hole pairs, resulting in enhanced photocatalytic activity [29]. However, there are few reports on the solution synthesis of BNQDs from small molecules and their composite preparation with ZnO for photocatalyst application. 

**Figure 1:**
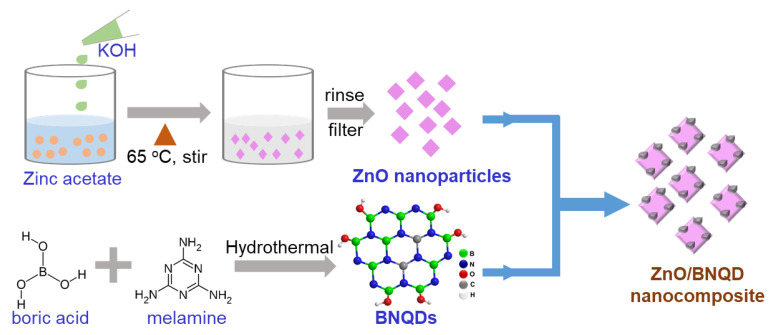
Schematic of the synthesis of ZnO/BNQD nanocomposites.

In this work, we present a facile method to synthesize a novel metal-free photocatalyst of BNQDs-decorated ZnO nanoparticles (ZnO/BNQD) to suppress the recombination of the photoinduced charges, which enhanced photocatalytic activity for the degradation of methylene blue (MB) and methyl orange (MO) in water with excellent stability. The BNQDs were synthesized through a facile hydrothermal process and the ZnO/BNQD composite was then prepared as shown in Figure 1. The amount of BNQD content in the ZnO/BNQD composite was varied and we investigated the photocatalytic performances for the degradation of MB and MO, which demonstrated the superior activities with the presence of BNQDs in ZnO. Furthermore, the ZnO/BNQD composite showed improved stability of the photocatalyst. The enhanced photocatalytic activity of the ZnO/BNQD nanocomposite is attributed to the heterojunction formation with excellent hole extraction by BNQDs and the suppressed recombination of photoinduced charge carriers. The detailed mechanism for photodegradation by the ZnO/BNQD composite is proposed with the radical scavenger tests. The present approach provides a promising alternative to photocatalysts for the degradation of organic dyes and environmental conservation.

## 2. Results and Discussion 

**Figure 2:**
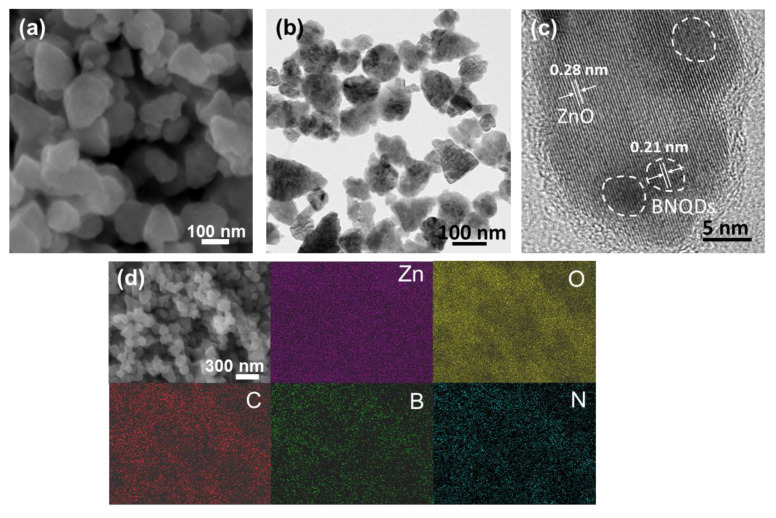
(**a**,**b**) FESEM and TEM images of the Zn/BNQD nanocomposite and (**c**) its high-resolution TEM image. (**d**) EDS mapping images of Zn/BNQD.

For the synthesis of BNQDs, boric acid and melamine molecules react and are converted into a boron nitride framework with oxygen functionalities at the edges of the BNQDs, providing high solubility and stability in aqueous media. The morphology of synthesized BNQDs was characterized by high-resolution transmission electron microscopy (HRTEM). The HRTEM image of BNQDs in Figure S1 shows that the obtained BNQDs are mostly spherical particles with an average size of approximately 4.6 nm. In the Raman spectrum of BNQDs (Figure S2a), a distinct peak at 1345 cm^−1^ is detected, which corresponds to the E_2g_ in-plane vibration mode of the boron and nitrogen atoms in the BN network. The chemical composition of BNQDs was characterized by X-ray photoelectron spectroscopy (XPS). The survey spectrum in Figure S2b displays clear peaks of O1s, N1s, C1s, and B1s, where the atomic ratios of BNQDs are 35. 4, 30.7, 19.5, and 14.4% for C, O, B, and N atoms, respectively. The presence of carbon atoms is primarily ascribed to the carbon atoms of melamine used as one of the reactants. For the ZnO/BNQD nanocomposites, Figure 2a shows the FESEM images of the prepared ZnO/BNQD sample, which consists of ZnO nanoparticles with an average size of 100 nm. Due to the small size of BNQDs, the morphology change in the ZnO/BNQD sample is not observed from the ZnO sample (Figure S3), where the average diameter of the synthesized ZnO particles is approximately 105 nm. TEM measurements were performed to characterize the morphology and microstructure of ZnO/BNQD. Figure 2b shows the deposition of BNQDs on the surface of ZnO nanoparticles. The nanodots of BNQDs in the high-resolution TEM image (Figure 2c) exhibit a spherical shape with an interplanar spacing value of 0.21 nm, which corresponds to the (100) facet of boron nitride. Meanwhile, the ZnO nanoparticle shows a lattice fringe of 0.28 nm, which is inconsistent with the (100) facet of ZnO. Moreover, an EDS elemental analysis was further carried out to observe the deposition and distribution of BNQDs on ZnO. Figure 2d shows the existence of Zn, O, C, B, and N elements distributed across the sample by the presence of BNQDs. Accordingly, these results could confirm the successful formation of the deposition of BNQDs on ZnO nanoparticles in the ZnO/BNQD nanocomposite. The crystal structure of ZnO nanoparticles and the ZnO/BNQD nanocomposite were characterized by XRD measurements. Figure S4 shows the XRD patterns of the ZnO nanoparticles and ZnO/BNQD nanocomposites with different BNQDs content. The diffraction peaks of ZnO for all samples correspond to a wurtzite structure of ZnO (JCPDS No. 36-1451) [23]. However, no different peaks or changes in peak positions are observed in the ZnO/BNQD nanocomposites, which is ascribed to the low content of BNQDs in the composite and relatively low crystalline structures of BNQDs compared to the high crystalline structure of ZnO. Furthermore, our bottom-up method using small molecules results in the synthesis of BNQDs with a few layers and large edge defects, which makes it difficult to obtain the characteristic XRD peaks of BNQDs. The chemical features of ZnO nanoparticles and the ZnO/BNQD nanocomposite were studied by FTIR measurement, as shown in Figure S5. The strong absorption peak at approximately 500 cm^−1^ corresponds to the stretching vibration mode of the Zn-O bond, and the absorption peaks at approximately 890 and 3450 cm^−1^ are assigned to the stretching of the C-H band and the hydroxyl group from the surface adsorbed species and H_2_O, respectively [30]. For the ZnO/BNQD nanocomposite, the group of new peaks in the range of 1100–1700 cm^−1^ is assigned to the typical stretching vibration modes of the C-N heterocycles [29], indicating that BNQDs have been successfully loaded on ZnO nanoparticles. 

**Figure 3:**
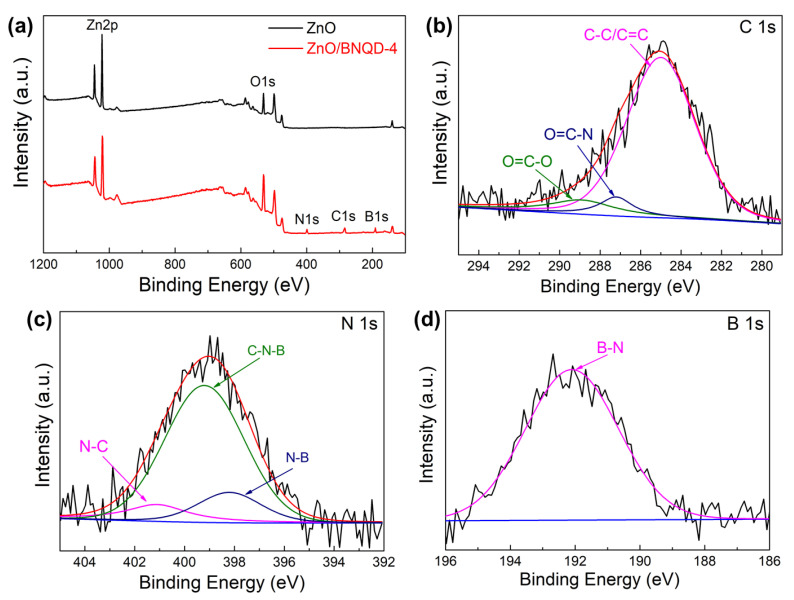
(**a**) XPS survey scan of ZnO and ZnO/BNQD-4 samples. (**b**–**d**) High-resolution C1s, N 1s, and Zn 2p spectra of ZnO/BNQD-4, respectively.

The XPS measurement was carried out to analyze the surface chemical composition and chemical states of the ZnO/BNQD nanocomposites. Figure 3a presents the survey scan of ZnO and ZnO/BNQD-4. It is evident that two elements of Zn and O are detected for ZnO, while three additional elements of B, C, and N are detected for the ZnO/BNQD by the presence of BNQDs. Figure 3b shows the high-resolution C1s spectrum of ZnO/BNQD-4, which can be divided into three peaks centered at 284.7, 287.4, and 289.1 eV, corresponding to the graphitic carbon (C-C/C=C), O=C-N, and O=C-O bonds, respectively. The high-resolution N1s spectrum (Figure 3c) is deconvoluted into three peaks centered at 398.2, 399.2, and 401.1 eV, which are assigned to N-B, C-N-B, and C-N-H bonds, respectively [29]. In the high-resolution B1s spectrum of ZnO/BNQD-4 (Figure 3d), the main peak is observed at 192.3 eV corresponding to B-N stretching vibration [25]. Additionally, the high-resolution XPS spectra of Zn 2p for ZnO and ZnO/BNQD-4 in Figure S6 exhibit two binding energy peaks of 1022.0 and 1045.1 eV for Zn 2p_3/2_ and Zn 2p_1/2_, respectively, showing the Zn^2+^ state of the prepared ZnO [31]. For ZnO/BNQD-4, it is observed that two characteristic peaks of Zn 2p shifted to 1021.5 and 1044.6 eV, which could be attributed to the interfacial interaction between ZnO and BNQDs.

**Figure 4:**
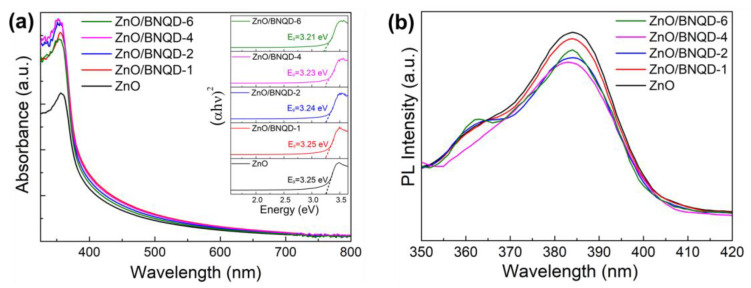
(**a**) UV-visible absorption spectra and (**b**) PL spectra of ZnO and different ZnO/BNQD nanocomposites. Inset showing the bandgap energy from UV-visible absorption spectra.

The optical properties of ZnO and ZnO/BNQD nanocomposites with different amounts of BNQDs were investigated by UV-visible diffuse reflectance spectroscopy as shown in Figure 4a. The ZnO exhibits distinct absorption in the UV region up to approximately 360 nm, corresponding to a band gap of 3.25 eV. The ZnO/BNQD samples show enhanced absorption in the visible range with increasing absorption intensity as the number of BNQDs increases. The corresponding band gap gradually decreases and is determined to be 3.25 eV (ZnO/BNQD-1), 3.24 eV (ZnO/BNQD-2), 3.23 eV (ZnO/BNQD-4), and 3.21 eV (ZnO/BNQD-6), which implies that the interaction between ZnO and BNQDs enhances the absorption toward the visible range and leads to an improvement in the visible light activity of the photocatalyst. The PL analysis was conducted to study the excitonic process related to the separation and transfer of photoinduced charge carriers. Figure 4b shows the PL emission spectra of the prepared photocatalysts at an excitation wavelength of 330 nm. ZnO exhibits the main emission peak at approximately 385 nm and displays the highest PL intensity. However, the ZnO/BNQD samples show lower PL intensities and the ZnO/BNQD-4 sample possesses the lowest PL intensity, suggesting the improved separation of photoinduced electron–hole pairs by the formation of a heterojunction between ZnO and BNQDs.

**Figure 5:**
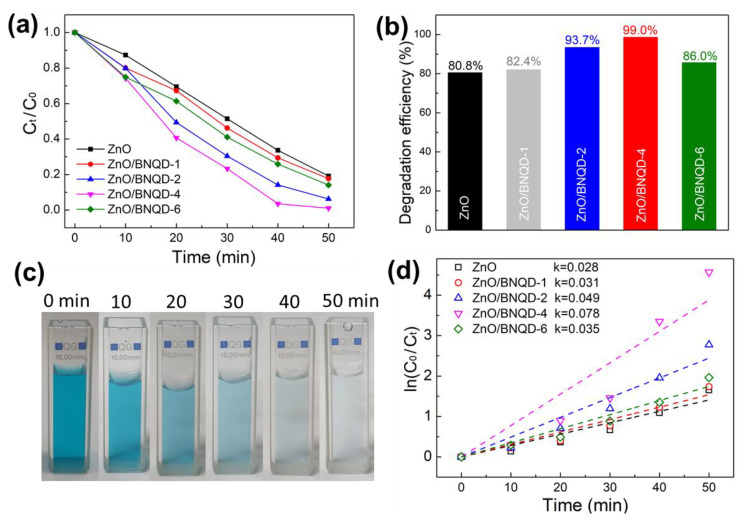
Photocatalytic degradation of MB; (**a**) plots of *C_t_*/*C*_0_ vs. UV irradiation time, (**b**) degradation efficiency, (**c**) a photograph of the color change of MB for different irradiation times decomposed by ZnO/BNQD-4, and (**d**) plots of *ln*(*C*_0_/*C_t_*) vs. irradiation time.

To evaluate the photocatalytic activities of the prepared ZnO/BNQD nanocomposites, the photodegradation test using methylene blue (MB) and methyl Orange (MO) was conducted under UV light irradiation. Before the UV irradiation, the photocatalysts were first mixed in the MB or MO solution for 1 h to ensure the adsorption/desorption equilibrium of MB or MO on the photocatalyst. As shown in Figure S7, the UV-vis spectrum reveals that the adsorption equilibrium for all photocatalysts was reached in 30 min. Thus, the photodegradation test was performed after 30 min of mixing. The UV-visible spectra for the photodegradation experiments of MB and MO solutions were recorded at different irradiation times for the ZnO and ZnO/BNQD nanocomposites (Figures S8 and S9). Figure 5a,b depict the degradation rate (*C_t_/C*_0_) and degradation efficiency of MB over the irradiation time for the ZnO and ZnO/BNQD nanocomposites, respectively. The ZnO exhibits relatively low photodegradation performances of 80.8% for MB after 50 min of irradiation, while the ZnO/BNQD photocatalysts exhibit significantly enhanced performances. Specifically, the ZnO/BNQD-4 photocatalyst delivers the highest photodegradation efficiency of 99% for MB. Further, Figure 5c indicates that the decolorization of MB dyes is completely delivered by the ZnO/BNQD-4 photocatalyst for the UV irradiation time. The rate constant (*k*) for the degradation of MB was further assessed by the following equation, where *t* is the irradiation time:

$$k=\frac{ln\;\left(C_{0}/C_{t}\right)}{t}$$

**Figure 6:**
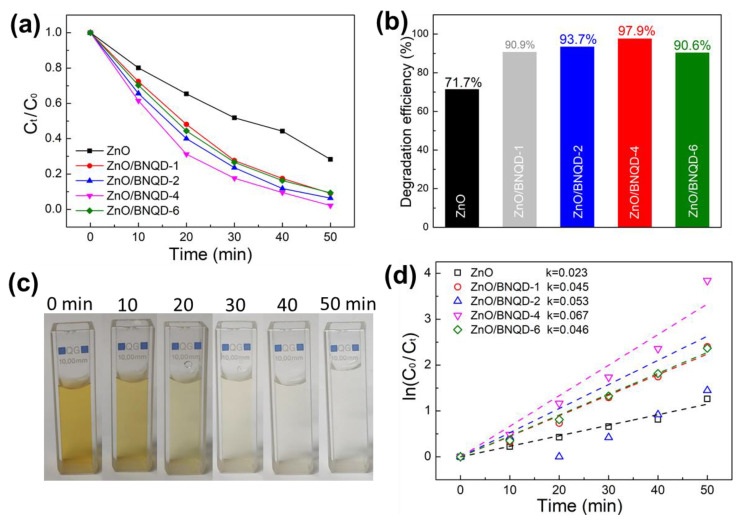
Photocatalytic degradation of MO; (**a**) plots of *C_t_*/*C*_0_ vs. UV irradiation time, (**b**) degradation efficiency, (**c**) a photograph of the color change of MO for different irradiation times decomposed by ZnO/BNQD-4, and (**d**) plots of *ln*(*C*_0_/*C_t_*) vs. irradiation time.

As displayed in Figure 5d, the plots of *ln* (*C*_0_/*C_t_*) for MB as the function of irradiation time (*t*) show a linear relationship, clarifying that the reaction of MB photocatalytic degradation coincides with the pseudo-first-order model. From the kinetic curves for MB photodegradation, the rate constant value (*k*) of ZnO, ZnO/BNQD-1, ZnO/BNQD-2, ZnO/BNQD-4, and ZnO/BNQD-6 are calculated to be 0.028, 0.031, 0.049, 0.078, and 0.035 min^−1^, respectively. Among the composites, ZnO/BNQD has the highest photocatalytic performance. The *k* value of ZnO/BNQD-4 is approximately 2.78 times as high as that of ZnO. Additionally, Figure 6a,b show the photodegradation performance for MO dyes. Similarly, the ZnO/BNQD photocatalysts exhibit an enhanced degradation performance compared to ZnO. The photodegradation efficiencies of ZnO/BNQD-1, ZnO/BNQD-2, ZnO/BNQD-4, and ZnO/BNQD-6 for MO are 90.9, 93.7, 97.9, and 90.6%, respectively, which show superior performance to that of ZnO (71.7%). The photographs of Figure 6c show the complete decolorization of MO for 50 min by ZnO/BNQD-4 photocatalyst. As shown in Figure 6d, the ZnO/BNQD-4 photocatalyst demonstrates the *k* value of 0.067 min^−1^, which is approximately 2.9 times higher than that of ZnO (0.023 min^−1^). Moreover, the ZnO/BNQD composites show superior performance for the photodegradation of MB and MO in comparison to the reported ZnO-based photocatalysts (Tables S1 and S2) [32,33,34,35,36,37]. This improved photocatalytic activity of ZnO/BNQD photocatalysts is attributed to the introduction of BNQDs suppressing the recombination of photoinduced electron–hole pairs. However, ZnO/BNQD-6 shows inferior photocatalytic activity compared to that of ZnO/BNQD-4, which is attributed to the excess presence of BNQDs covering the active sites of ZnO, resulting in the decrease in light absorption, in agreement with the PL results. 

**Figure 7:**
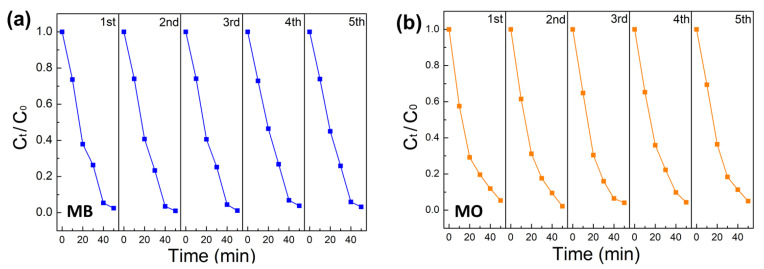
The cyclic stability of ZnO/BNQD-4 for five cycles: (**a**) In MB and (**b**) in MO.

Moreover, the stability of the ZnO/BNQD-4 photocatalyst was investigated by conducting cyclic experiments in the photocatalytic degradation of MB and MO (Figure 7). It is evident that the stable photocatalytic activity of ZnO/BNQD-4 was achieved for the photodegradation of MB and MO with no significant deterioration for five successive cycles. These results confirm that the prepared ZnO/BNQD photocatalysts possess excellent stability for the photodegradation of MB and MO dyes.

**Figure 8:**
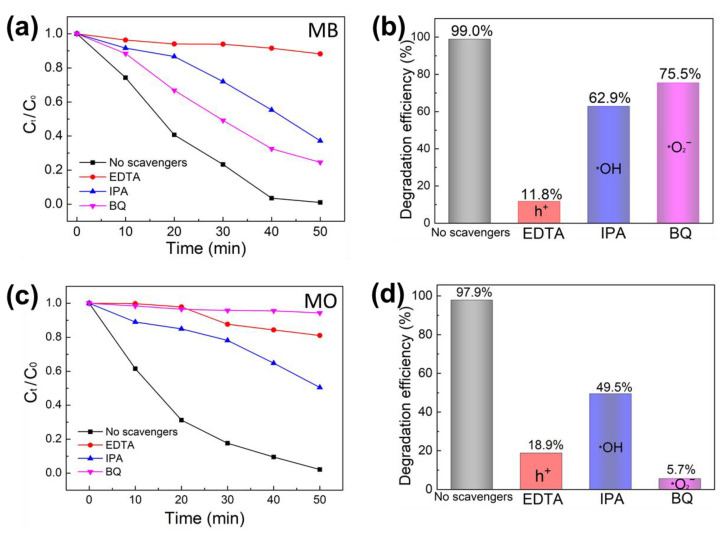
Effects of different scavengers on the photocatalytic degradation and degradation efficiency with ZnO/BNQD-4 nanocomposite for (**a**,**b**) MB and (**c**,**d**) MO.

Figure 8 depicts the different active species trapping results for the photodegradation of MB and MO using ZnO/BNQD-4 photocatalyst. Herein, EDTA, IPA, and BQ were employed as the scavengers of h^+^, ·OH, and ·O_2_^−^, respectively [14]. Figure 8a,b show the degradation rate and efficiencies for the photodegradation of MB dyes, respectively. With the presence of BQ and IPA, the degradation efficiency of MB is reduced from 99% to 75.5 and 62.9% due to the removal of ·OH and ·O_2_^−^ by IPA and BQ, respectively. However, the addition of EDTA led to a distinct decrease in the degradation efficiency of MB from 99 to 11.8%, which indicates that h^+^ plays a bigger leading role than ·O_2_^−^ or ·OH in the photodegradation of MB. In the case of the photodegradation of MO dyes (Figure 8c,d), the addition of EDTA, IPA, and BQ results in the apparent reduction of photodegradation efficiency from 97.9 to 18.9, 49.5, and 5.7%, respectively. These results suggest that these three active species (h^+^, ·OH, and ·O_2_^−^) played important roles in the photodegradation of MO, and among them, ·O_2_^−^ and h^+^ are the major reactants. 

**Figure 9:**
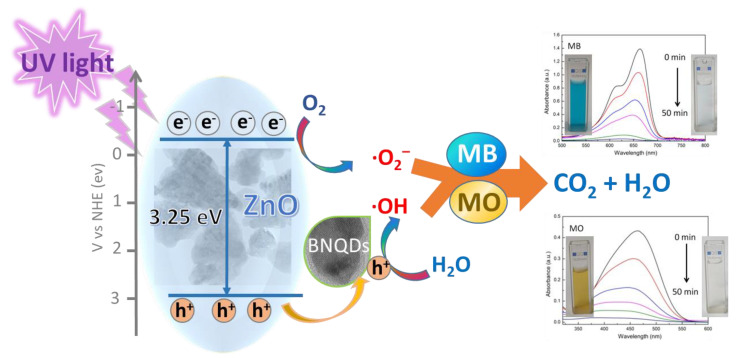
Schematic of the possible photocatalytic mechanism of ZnO/BNQD nanocomposites for MB and MO degradation.

The photocatalytic mechanism of ZnO/BNQD nanocomposites is proposed in Figure 9. Under UV irradiation, the photoinduced holes (*h^+^*) in the valence band of ZnO (+2.94 eV) are extracted by BNQDs, resulting in the efficient separation of photoinduced electrons and holes in the photocatalysts. Therefore, the BNQDs with the extracted holes (*h^+^*) from ZnO can generate reactive hydroxyl radicals (·OH) by the reaction with the absorbed water molecules, while the photoinduced electrons in the conduction band of ZnO (−0.31 eV) can react with the trapped oxygen molecules to generate superoxide radicals (·O_2_^−^). Finally, the generated active species ( ·OH and ·O_2_^−^) effectively oxidize organic dyes of MB and MO in the photodegradation process, which is consistent with the above results.

## 3. Experimental

### 3.1. Preparation of ZnO Nanoparticles

To synthesize ZnO nanoparticles, 5.2 g of zinc acetate dehydrate (Zn(CH_3_COO)_2_·2H_2_O) was first dissolved in 200 mL of deionized (DI) water at 65 °C under constant stirring. Simultaneously, 100 mL of 0.5 M KOH solution was added dropwise to a zinc acetate dehydrate solution under vigorous stirring at 65 °C, and after 1 h of stirring, a white-colored milky suspension was obtained. After centrifugation of the suspension mixture, the obtained product was then filtered and rinsed with ethanol several times to remove the residue organic materials and dried at 100 °C in an oven.

### 3.2. Preparation of BNQDs

The BNQDs were synthesized through a hydrothermal process. In detail, 100 mg of boric acid and 34 mg of melamine were dissolved in 10 mL of DI water with constant stirring. The prepared suspension was transferred into a Teflon-lined autoclave and kept at 200 °C for 15 h. After cooling to room temperature, the resulting solution mixture was dialyzed using a dialysis tube (3000 Da, Spectrum Lab. Inc., Milpitas, U.S.) against DI for 2 days for the removal of unreacted chemicals and impurities to obtain the BNQDs solution. The final BNQDs solution was obtained with a concentration of 3 mg/mL.

### 3.3. Preparation of ZnO/BNQD Composites

To prepare ZnO/BNQD composites, 0.3 g of ZnO nanoparticles was firstly dispersed in 50 mL of ethanol and sonicated for 1 h. Subsequently, a certain amount of the BNQDs solution was added dropwise to the ZnO suspension and stirred for 24 h. Finally, after the ethanol was completely vaporized, the ZnO/BNQD composites were collected and dried in a vacuum oven at 70 °C. The obtained composites with different BNQD contents are designated as ZnO/BNQD-x, where “x” denotes the weight ratio of the added BNQDs (x = 1, 2, 4, and 6 wt.%).

### 3.4. Characterization

X-ray diffraction (XRD) measurement was performed using a D8 Advance diffractometer (Bruker, Billerica, U.S.) with a non-monochromated Cu-Kα operated at 40 kV and 30 mA. The morphology and energy-dispersive X-ray spectroscopy (EDS) element mapping of the products were collected by a field-emission scanning electron microscope (FE-SEM, JEOL-JSM820, JEOL, Tokyo, Japan). Transmission electron microscopy (TEM; H-8100, Hitachi, Tokyo, Japan) was carried out at an accelerating voltage of 200 kV. X-ray photoelectron spectroscopy (XPS; K-alpha spectrometer, Thermo Fisher, Waltham, U.S.) measurements were performed to analyze the surface chemical composition. Fourier transform infrared (FTIR) spectra were collected by the Thermal Fisher Nicolet iS5 spectrometer. Ultraviolet visible (UV-vis) diffuse reflectance spectra were recorded by the Specord-210-Plus spectrophotometer. The photoluminescence (PL) spectra were recorded by the Agilent Technologies PCB-1500 spectrophotometer at 330 nm excitation wavelength. 

### 3.5. Photocatalytic Degradation Measurement

The photocatalytic activities of the obtained ZnO/BNQD photocatalysts were examined using degradation experiments of MB or MO under UV light irradiation. Four UV bench lamps (Sigma black light bulb, 20 W, 315~400 nm) were employed as UV light sources. In a typical photocatalytic degradation experiment, 50 mg of the prepared photocatalyst powder was added into 50 mL of an aqueous solution of MB or MO dyes (10 mg/L). Before illumination, the suspension was stirred in the dark for 30 min to reach the adsorption/desorption equilibrium of dyes on the surface of the photocatalyst. Then, the prepared suspension was positioned at a distance of 15 cm from the UV lamps for different irradiation times, and 3 mL of the reaction solution was collected every 10 min and filtered to separate catalysts. The reaction solution kept stirring during the irradiation in order to prevent uncertainties from the measurements and the sampling errors. The concentrations of dyes in the tested solution were characterized by UV-vis spectroscopy from the absorption peaks of the MB and MO dyes at 665 and 470 nm, respectively. The photocatalytic degradation efficiency (*DE*%) of dyes was also calculated by the following equation:

$$DE\left(\%\right)=\frac{C_{0}-C_{t}}{C_{0}}\times 100$$

where *C*_0_ represents the initial concentration of dye, and *C_t_* is the concentration of dye after photodegradation.

To further understand the photocatalytic degradation mechanism, active species trapping experiments were conducted to determine the dominant active species during the photodegradation of MB and MO dyes. The trapping agents, including ethylenediamine tetraacetate (EDTA), 1,4-benzoquinone (BQ), and isopropanol (IPA) were employed as scavengers to trap the photo-excited holes (h^+^), superoxide radical (·O_2_^−^) and hydroxyl radical (·OH), respectively. 

## 4. Conclusions

In summary, the ZnO/BNQD nanocomposites were successfully prepared by a facile method and their photocatalytic activities were tested for the photodegradation of MB and MO under UV irradiation. The prepared ZnO/BNQD nanocomposites demonstrated significantly enhanced performance for the photodegradation of MB and MO with excellent stability. This result is attributed to the efficient extraction of photoinduced holes from ZnO toward BNQDs, which suppresses the recombination of photoinduced charge to generate the active species for the degradation of MB and MO dyes. Thus, the ZnO/BNQD nanocomposites in this work show potential for efficient metal-free photocatalysts for environmental remediation and energy conversion. 

## Data Availability

The data presented in this study are available upon request from the corresponding author.

## Supplementary Materials

The following supporting information can be downloaded at: https://www.mdpi.com/article/10.3390/molecules27206833/s1, Figure S1: HRTEM image and the size distribution of the synthesized BNQDs; Figure S2: Raman spectrum and XPS survey scan of BNQDs; Figure S3: FESEM image and the size distribution of the synthesized ZnO particles; Figure S4: XRD patterns of ZnO and ZnO/BNQD nanocomposites; Figure S5: FTIR spectra of ZnO and ZnO/BNQD-4 nanocomposite; Figure. S6: High resolution XPS spectra of Zn 2p for ZnO and ZnO/BNQD-4 nanocomposite; Figure S7: The adsorption equilibrium for ZnO and ZnO/BNQD-4 nanocomposite in MB and MO; Figure S8: UV-vis spectra of MB solution with ZnO and ZnO/BNQD nanocomposites at different irradiation times; Figure S9: UV-vis spectra of MO solution with ZnO and ZnO/BNQD nanocomposites at different irradiation times; Table S1: Photocatalytic activities comparison for the degradation of MB with ZnO based composites under UV light irradiation; Table S2: Photocatalytic activities comparison for the degradation of MO with ZnO based composites under UV light irradiation.
