# Supplementary material for: ZnO/Boron Nitride Quantum Dots Nanocomposites for the Enhanced Photocatalytic Degradation of Methylene Blue and Methyl Orange
Source: Molecules. 2022 Oct 12;27(20):6833. doi: 10.3390/molecules27206833 (PMC9612324; doi:10.3390/molecules27206833)
Supplement: Supplementary file 1 [file molecules-27-06833-s001.zip › molecules-1946687-supplementary.pdf]

# Supporting Information

# ZnO/boron nitride quantum dots nanocomposites for the enhanced photocatalytic degradation of methylene blue and methyl orange

Di Liu†, Jinu Song†, Jin Suk Chung, Seung Hyun Hur*, Won Mook Choi*

*School of Chemical Engineering, University of Ulsan, 93 Daehak-ro Nam-gu, Ulsan 44610, Korea*

†These authors contributed equally to this work.

*Corresponding author.

Tel.: +82 52 259 1065, fax: +82 52 259 1689

E-mail addresses: shhur@ulsan.ac.kr (S.H. Hur)

wmchoi98@ulsan.ac.kr (W.M. Choi)

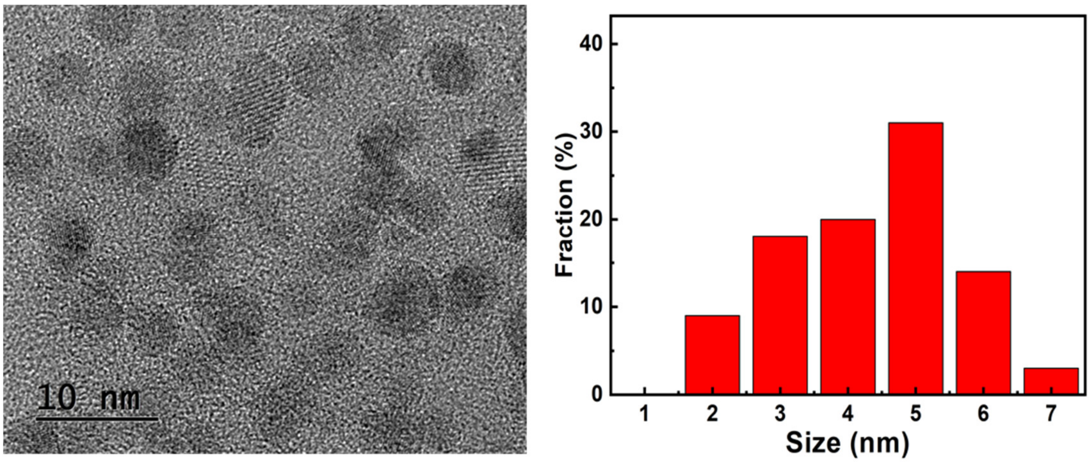


**Figure S1**. HRTEM image and the size distribution of the synthesized BNQDs.

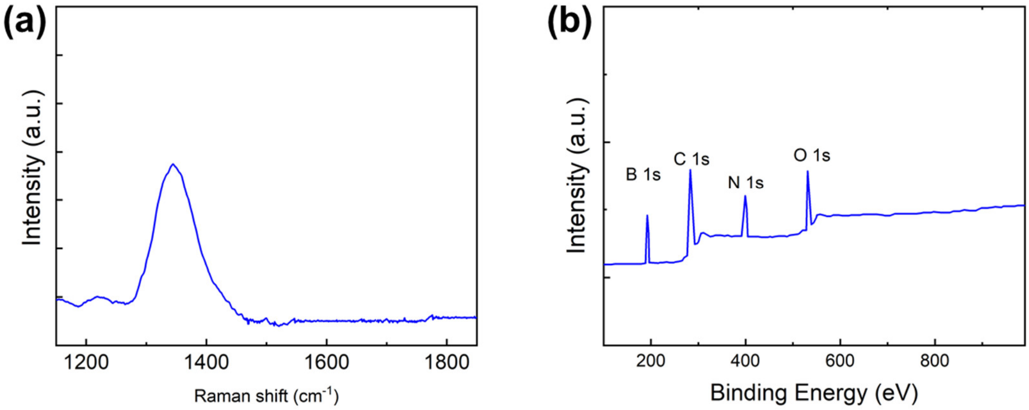


**Figure S2.** (a) Raman spectrum and (b) XPS survey scan of BNQDs.

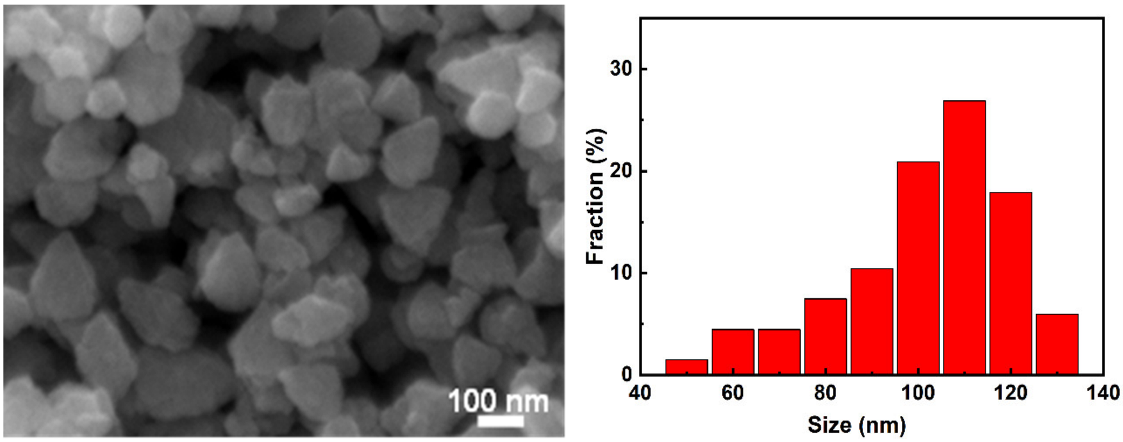


**Figure S3**. FESEM image and the size distribution of the synthesized ZnO particles.

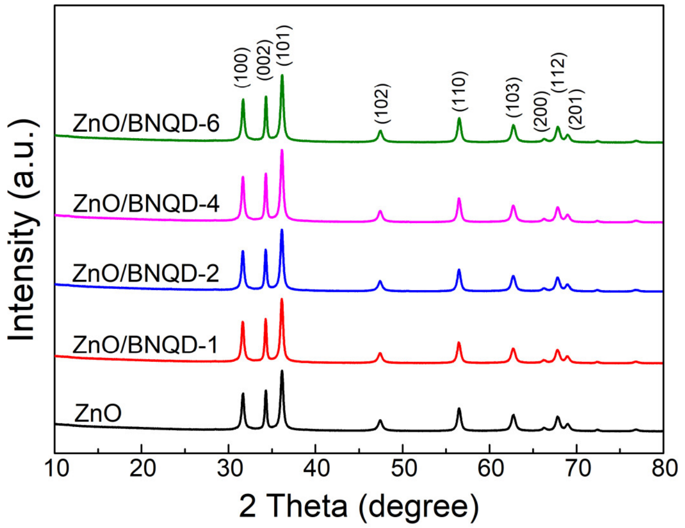


**Figure S4**. XRD patterns of ZnO and ZnO/BNQD nanocomposites.

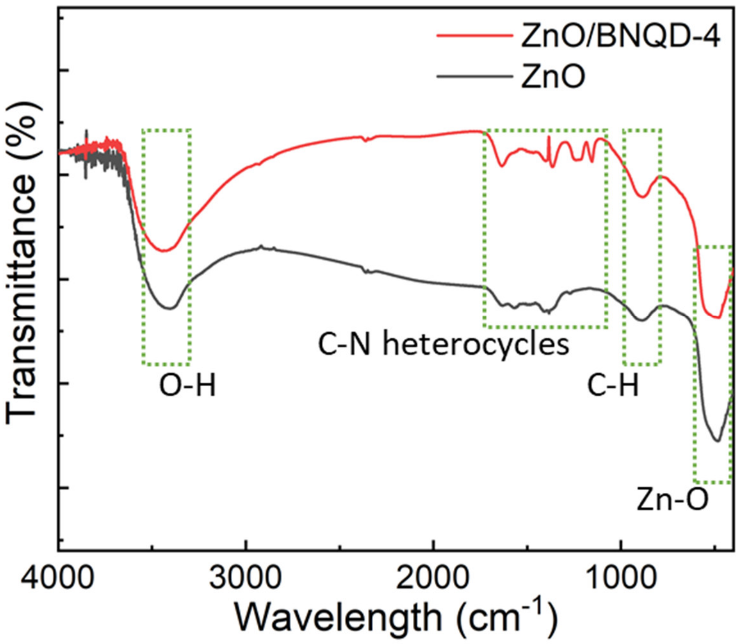


**Figure S5**. FTIR spectra of ZnO and ZnO/BNQD-4 nanocomposite.

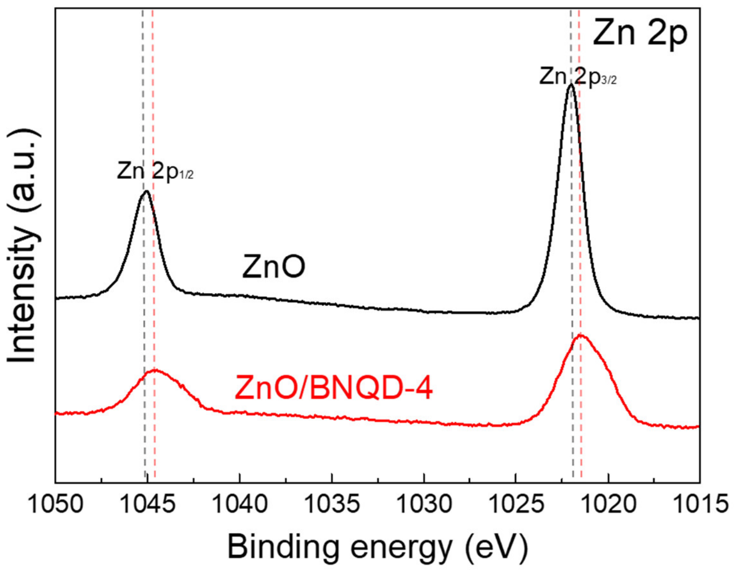


**Figure. S6**. High resolution XPS spectra of Zn 2p for ZnO and ZnO/BNQD-4 nanocomposite.

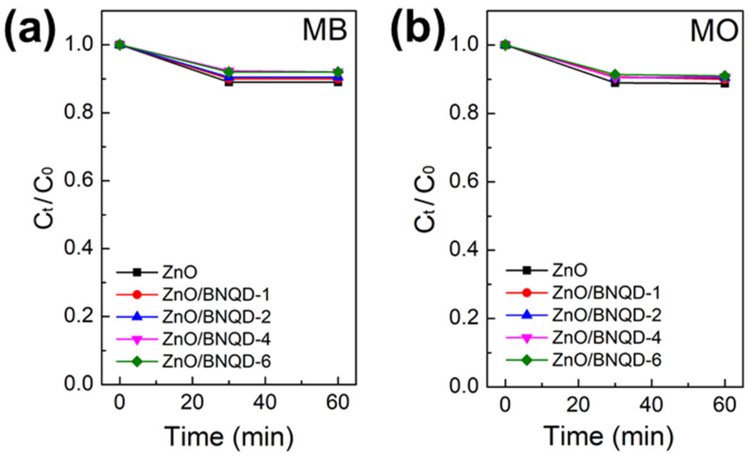


**Figure S7**. The adsorption equilibrium for ZnO and ZnO/BNQD-4 nanocomposite in (a) MB and (b) MO.

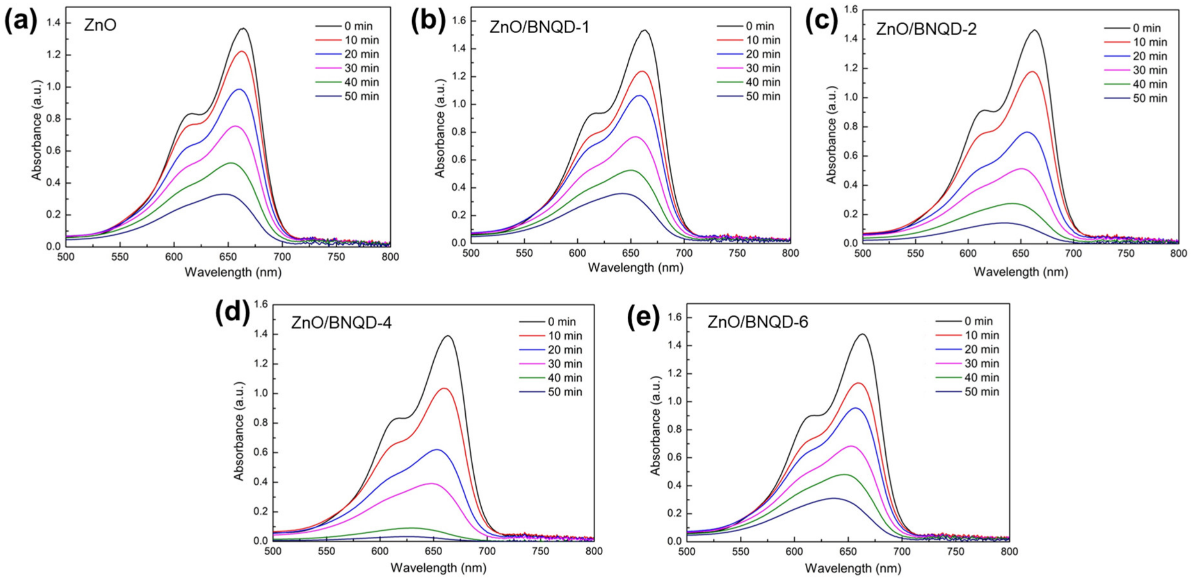


**Figure S8**. UV-vis spectra of MB solution with (a) ZnO and (b-e) ZnO/BNQD nanocomposites at different irradiation times.

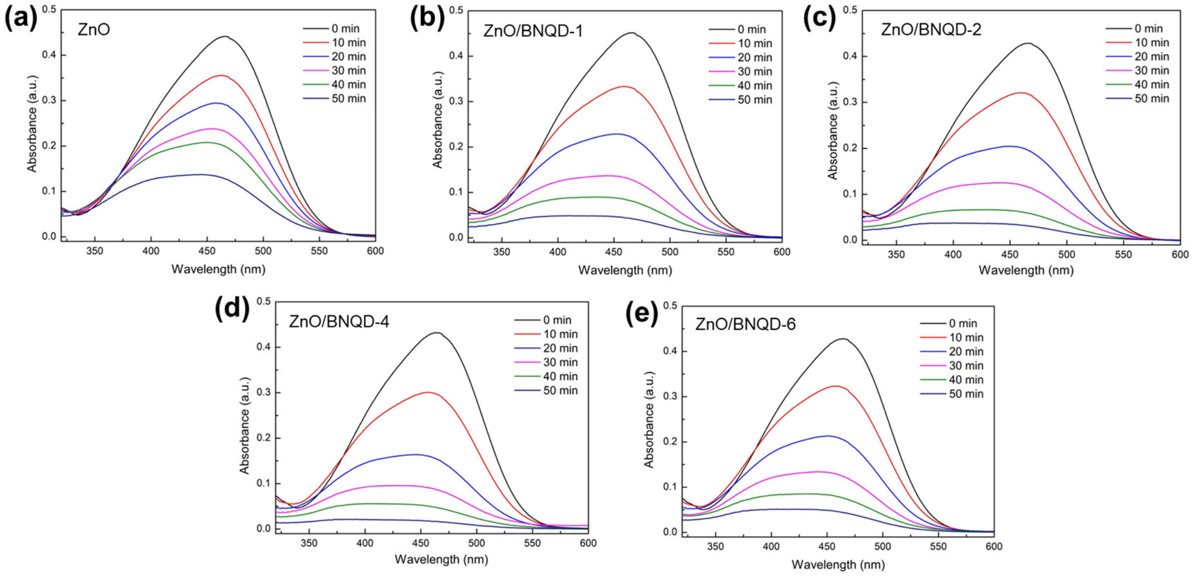


**Figure S9**. UV-vis spectra of MO solution with (a) ZnO and (b-e) ZnO/BNQD nanocomposites at different irradiation times.

Table S1. Photocatalytic activities comparison for the degradation of MB with ZnO based composites under UV light irradiation.

| Sample | Catalyst dosage (g/L) | Light source | Irradiation time | MB degradation (%) | Rate constant ($min^{-1}$) | Ref. |
|---|---|---|---|---|---|---|
| N-doped ZnO/$C_3N_4$ | 0.5 g/L | 300W Xe lamp | 100 min | 90% | 0.0299 | [1] |
| Ag/ZnO | 0.6 g/L | 6W UV lamp | 60 min | 87.74% | 0.032 | [2] |
| ZnO/Eu | 1 g/L | 300W Osram Vitalux | 150 min | 90% | - | [3] |
| N-ZnO/$C_3N_4$ | 1 g/L | 300W Xe lamp | 90 min | 95% | 0.030 | [4] |
| AgBr/g-$C_3N_4$/ZnO | 0.4 g/L | 300W Xe lamp | 80 min | 96.3% | 0.041 | [5] |
| N self doped ZnO | 0.5 g/L | 200W Xe lamp | 80 min | 95.3% | 0.040 | [6] |
| ZnO/BNQD | 1 g/L | 20W Sigma 4 BLB | 40 min | 96.4% | 0.0776 | This work |

Table S2. Photocatalytic activities comparison for the degradation of MO with ZnO based composites under UV light irradiation.

| Sample | Catalyst dosage (g/L) | Light source | Irradiation time | MO degradation (%) | Rate constant ($min^{-1}$) | Ref. |
|---|---|---|---|---|---|---|
| ZnO/Eu | 1.0 g/L | 300 W Osram Vitalux | 60 min | 95% | - | [3] |
| Graphene/ZnO | 0.5 g/L | Natural sunlight | 150 min | 100% | 0.035 | [7] |
| Ag-N-ZnO | 0.2 g/L | 500 W Xe lamp | 120 min | 98.82% | 0.026 | [8] |
| $ZnO/SnO_2$ | 0.2 g/L | 300 W Hg lamp | 100 min | 56% | - | [9] |
| $Cu\text{-}TiO_2/ZnO$ | 1.0 g/L | 500 W Xe lamp | 60 min | 83% | 0.0306 | [10] |
| $ZnO/g\text{-}C_3N_4$ | 2.5 g/L | 500 W Xe lamp | 120 min | 98% | 0.136 | [11] |
| ZnO/BNQD | 1.0 g/L | 20W Sigma 4 BLB | 50 min | 97.9% | 0.0666 | This work |

# References

[1] Balcha, A.; Yadav, O.P.; Dey, T. Photocatalytic degradation of methylene blue dye by zinc oxide nanoparticles obtained from precipitation and sol–gel methods. *Environ. Sci. Pollut. Res.* **2016**, *23,* 25485–25493.

[2] Rafaie, H.A.; Nor, R.M.; Azmina, M.S.; Ramli, N.I.T.; Mohamed, R. Decoration of ZnO microstructures with Ag nanoparticles enhanced the catalytic photodegradation of methylene blue dye. *J. Environ. Chem. Eng.* **2017**, *5,* 3963–3972.

[3] Trandafilović, L.V.; Jovanović, D.J.; Zhang, X.; Ptasińska, S.; Dramićanin, M.D. Enhanced photocatalytic degradation of methylene blue and methyl orange by ZnO:Eu nanoparticles. *Appl. Catal. B: Environ*. **2017**, *203*, 740–752.

[4] Liu, Y.; Liu, H.; Zhou, H.; Li, T.; Zhang, L. A Z-scheme mechanism of N-ZnO/g-$C_3N_4$ for enhanced $H_2$ evolution and photocatalytic degradation. *Appl. Surf. Sci*. **2019**, *466*, 133–140.

[5] Azimi, E.B.; Badiei, A.; Sadr, M.H. Dramatic visible photocatalytic performance of g-$C_3N_4$-based nanocomposite due to the synergistic effect of AgBr and ZnO semiconductors. *J. Phys. Chem. Solids.* **2018**, *122*, 174–183.

[6] Sun, L.; Shao, Q.; Zhang, Y.; Jiang, H.Y.; Ge, S.S.; Lou, S.Q.; Lin, J.; Zhang, J.X.; Wu, S.D.; Dong, M.Y.; Guo, Z.H. N self-doped ZnO derived from microwave hydrothermal synthesized zeolitic imidazolate framework-8 toward enhanced photocatalytic degradation of methylene blue. *J. Colloid Interface Sci.* **2020**, *565*, 142–155.

[7] Wang, L.; Li, Z.; Chen, J.; Huang, Y.; Zhang, H.; Qui, H. Enhanced photocatalytic degradation of methyl orange by porous graphene/ZnO nanocomposite. *Environ. Pollut.* **2019**, *249,* 801–811.

[8] Chen, X.; Wu, Z.; Gao, Z.; Ye, B.C. Effect of different activated carbon as carrier on the photocatalytic activity of Ag-N-ZnO photocatalyst for methyl orange degradation under visible light irradiation. *Nanomaterials* **2017**, *7*, 258.

[9] Ali, W.; Ullah, H.; Zada, A.; Alamgir, M.K.; Muhammad, W.; Ahmad, M.J.; Nadhman, A. Effect of calcination temperature on the photoactivities of ZnO/$SnO_2$ nanocomposites for the degradation of methyl orange. *Mater. Chem. Phys.* **2018**, *213*, 259–266.

[10] Dorraj, M.; Alizadeh, M.; Sairi, A.; Basirun, W.; Goh, B.T.; Woi, P.; Alias, Y. Enhanced Visible Light Photocatalytic Activity of Copper-doped Titanium Oxide–Zinc Oxide Heterojunction for Methyl Orange Degradation. *Appl. Surf. Sci.* **2017**, *414*, 251–261.

[11] Guan, R.; Li, J.; Zhang, J.; Zhao, Z.; Wang, D.; Zhai, H.; Sun, D. Photocatalytic Performance and Mechanistic Research of ZnO/g-$C_3N_4$ on Degradation of Methyl Orange. *ACS omega* **2019**, *4*, 20742–20747.
